# Rapid Adaptation of HIV Treatment Programs in Response to COVID-19 — Namibia, 2020

**DOI:** 10.15585/mmwr.mm6942a6

**Published:** 2020-10-23

**Authors:** Steven Y. Hong, Laimi S.N. Ashipala, Leonard Bikinesi, Ndapewa Hamunime, Jacques W.N. Kamangu, Ashley Boylan, Edwin Sithole, Ismelda C. Pietersen, Gram Mutandi, Catherine McLean, Eric J. Dziuban

**Affiliations:** ^1^Division of Global HIV and TB, Center for Global Health, CDC; ^2^Ministry of Health and Social Services, Namibia.

Namibia is an upper-middle income country in southern Africa, with a population of approximately 2.5 million (*1*). On March 13, 2020, the first two cases of coronavirus disease 2019 (COVID-19) in Namibia were identified among recently arrived international travelers. On March 17, Namibia’s president declared a state of emergency, which introduced measures such as closing of all international borders, enactment of regional travel restrictions, closing of schools, suspension of gatherings, and implementation of physical distancing measures across the country. As of October 19, 2020, Namibia had reported 12,326 laboratory-confirmed COVID-19 cases and 131 COVID-19–associated deaths. CDC, through its Namibia country office, as part of ongoing assistance from the U.S. President’s Emergency Plan for AIDS Relief (PEPFAR) provided technical assistance to the Ministry of Health and Social Services (MoHSS) for rapid coordination of the national human immunodeficiency virus (HIV) treatment program with the national COVID-19 response.

With support from PEPFAR since 2004, Namibia is on the verge of HIV epidemic control: 95% of persons with HIV infection know their status; 95% of these persons are receiving antiretroviral therapy (ART); and among these, 92% have achieved viral load suppression (≤1,000 copies of viral RNA/mL) (*2*). Because the COVID-19 pandemic has the potential to compromise Namibia’s ART program efforts, MoHSS prioritized providing life-saving ART while reducing patient volume in ART facilities to reduce the risk for COVID-19 exposure in advance of a possible broader COVID-19 outbreak in Namibia.

Regional MoHSS ART clinical mentors, who are experienced ART physicians supporting healthcare workers in each of the 14 regions, served as points of contact to implement rapid adjustments to the ART program. New national guidance, coordination, and feedback were communicated through the Project Extension for Community Healthcare Outcomes (ECHO) virtual mentoring platform (*3*); Namibia was among the first African countries to adopt Project ECHO in 2015. During March 17–April 21, MoHSS conducted seven communication sessions using the Project ECHO platform with 760 sites and 2,068 health care providers. Because all major district hospitals and high-volume health care centers in the country use the ECHO platform, rapid communication and telementoring across all regions was possible, despite travel restrictions.

MoHSS, with CDC support and in alignment with forthcoming PEPFAR guidance (*4*), quickly developed a plan to minimize the frequency of patient contact with the health care system and reduce burden on facilities. The plan consisted of facility readiness, multimonth dispensing (MMD) of ART, and the expansion of community ART dispensing.

Facility readiness included plans for screening and triaging patients. ART patients were first screened for COVID-19–compatible signs and symptoms* upon arrival at the health facility. Those with symptoms were isolated and tested for SARS-CoV-2, the virus that causes COVID-19, by polymerase chain reaction (PCR) testing of specimens obtained with a nasopharyngeal swab and, if hospitalization was not required, were asked to self-isolate while waiting for results. In an effort to avoid overcrowded waiting areas, and thereby possible SARS-CoV-2 transmission, asymptomatic patients were triaged to receive fast-track refills without entering the facility or quick, small group clinical consultations for dispensing MMD. Patients aged ≥50 years and those with underlying medical conditions (*5*) received expedited services. MoHSS provided recommended personal protective equipment (*6*) to clinic staff members and symptomatic patients.

Four-month MMD of ART was implemented by assessing the national stock and adjusting ART guidance to maximize available stock and ensure optimal regimens. To ensure treatment continuity, the National Central Medical Store distributed 4–6 months’ supply of stock for 166,237 (97%) of 171,830 total patients receiving ART to health care facilities in all regions. Emergency procurement was activated to ensure that a 12-month supply of ART stock would be available in the country.

Community ART dispensing was expanded through 1) newly established community-based ART points, 2) primary health care outreach points, 3) community adherence groups, 4) mobile vans, and 5) home delivery. Outreach points placed at the borders were especially important for Angolan patients seeking ART refills in Namibia despite border closures. A national ART hotline was established to assist patients who experienced difficulty accessing services.

Other HIV treatment services were also adjusted to prevent transmission of SARS-CoV-2 (Table). Programs minimized patient contact with health care facilities to limit possible exposure. Community programming supported physical distancing and used alternative methods of communication, including virtual platforms such as Zoom or Skype, phone calls, social media, and WhatsApp Messenger, a mobile application for smartphones. Group activities were limited in size according to Namibia national regulations.

**TABLE:** Adaptations of the national human immunodeficiency virus (HIV) testing and treatment program during the COVID-19 outbreak — Namibia, 2020

Program area	Program adaptations
**Clinic readiness**	Screening and triaging clients for COVID-19 symptoms
	Adjusting clinic flow to limit overcrowding and exposure risk for patients and health care workers
	Limiting clinic appointments to avoid crowding (prioritizing patients failing ART or clinical complaints)
	Providing ART refills without entering the facility
	Providing expedited services for patients at higher risk for COVID-19 morbidity and mortality
	Using Project ECHO platform for regular communication, coordination and telementoring for all regions
	Providing virtual COVID-19 trainings for staff members
	Providing PPE
**MMD of ART (adults, children, and pregnant and breastfeeding women)**	Assessing national stock situation and adjusting ART guidance to ensure adequate medication stock for MMD
	Issuing and widely sharing interim ART guidance through ECHO and clinical mentorship network
	Distributing stock to regions to ensure adequate supplies for MMD
**Community dispensing**	Using community-based ART outreach points
	Adapting and expanding community adherence groups with groups <10 at one time
	Expanding primary health care outreach points
	Using mobile vans (especially for persons at high risk and for those unable to get MMD)
	Establishing home delivery (through community health care workers)
**Patient tracing**	Shifting physical tracing of patients missing appointments to phone tracing exclusively
**Strengthening border services**	Liaising with regional governments, immigration authorities, and police to allow patients to access medicines
	Creating outreach points for patients to access ART at the Angola-Namibia border
	Tracing patients who miss appointments via telephone to link them back into care
	Establishing ART hotline to assist patients having difficulty accessing services and answer questions about COVID-19 and HIV
**Facility HIV testing**	Prioritizing facility-based testing (antenatal care, admitted patients, early infant diagnosis, persons with tuberculosis and sexually transmitted infections, passive index testing)
	Standardizing safe HIV testing services provision using physical distancing and PPE measures
	Maximizing use of self-testing kits* outside of clinic settings for clients and their partners
**Community index testing**	Discontinuing community index testing until safe processes were established
	Providing guidance for proper PPE use to implement community index testing once deemed safe
**HIV recency testing**	Pausing recency testing to decrease time spent in clinics and decrease burden on laboratory staff members
**Community adolescent treatment supporters and teen clubs**	Providing community adolescent treatment supporters with mobile phones and airtime to continue to engage beneficiaries from home
	Limiting teen clubs to continue meeting in only places where they could practice recommended physical distancing with no more than 10 teens at a time
**Cervical cancer screening and treatment**	Continuing limited screening at health facilities that have program-specific staff members
	Postponing all outreach and campaigns
**Tuberculosis preventive therapy**	Dispensing tuberculosis preventive therapy for the full duration of treatment for those initiating or already receiving tuberculosis preventive therapy
	Delivering tuberculosis preventive therapy medications to eligible clients through community health care workers with recommended PPE and physical distancing
**Prevention of mother-to-child transmission of HIV**	Continuing routine testing of pregnant and breastfeeding women and HIV-exposed infants
	Providing MMD for pregnant and breastfeeding women
	Prioritizing safe labor and delivery access in health care facilities with adjustments in clinic flow to minimize the risk for COVID-19 exposure
	Providing pregnant and breastfeeding women adherence and retention support through telephone calls

**Abbreviations:** ART = antiretroviral therapy; COVID-19 = coronavirus disease 2019; MMD = multimonth dispensing; PPE = personal protective equipment; Project ECHO = Extension for Community Healthcare Outcomes.

* OraSure Technologies. http://www.oraquick.com.

Namibia has rapidly implemented public health measures to mitigate SARS-CoV-2 transmission, which allows additional time to adequately prepare the health care system for a potential surge in COVID-19 cases. The ART program has adapted to ensure the continuity of essential HIV services while maintaining a safe health care environment for clients and staff members during the COVID-19 pandemic. Efforts are underway to evaluate the implementation of these initiatives across sites and the impact on programs. These public health strategies could be implemented in other settings where COVID-19 might threaten the HIV treatment program when the public health providers and governments are willing to use new technologies and novel strategies to maintain patient care.

## Footnotes

* Fever or temperature ≥100.4°F (38.0°C), cough, shortness of breath, weakness, muscle aches, chills, vomiting or diarrhea, headache, chest pain, new loss of taste or smell.
